# Errata

**Published:** 1881-12

**Authors:** 


### ERRATA.

ERRATA in Dr. Rendell's proving of *Am. carb.*, page 292-305:

Symptom 20-21, these should be united as one symptom.

" 33, for *pains* read *pain*, (also in symptoms 78, 151, 157).

" 45, the last two paragraphs, commencing with "smarting," should be given as distinct symptoms.

" 69, for *lips* read *lip*.

" 102, for first *would* read *could*.

" 111, for *stooping* read *stepping*.

" 123, for *tenderness* read *tenesmus*; for *during* read *During*.

" 138, for *groins* read *groin*.

" 151, last line; place full stop after "evening;" in first line on page 302, for *when* read *When*, and place comma instead of a semicolon after "better."

" 157, for *stopping* read *stooping*.

" 171, for *arms* and *elbows* read *arm* and *elbow*.

SKIN: Add. "Red rash on chest, very thick and burning, (32 to 39.)"

ERRATA in Dr. Wells' "Diphtheria and Bacteria," etc:

Page 548, 12th line from bottom, for *Hauft* read *Haupt*.

" 548, 2d line from bottom, for *eminent* read *earnest*.

" 549, 7th line from bottom for *generously* read *seriously*.

